# Leptin and Leptin Receptor Polymorphisms in Infants and Their Parents: Correlation with Preterm Birth

**DOI:** 10.3390/genes15010139

**Published:** 2024-01-22

**Authors:** Francesco Savino, Allegra Sardo, Stefano Gambarino, Maddalena Dini, Anna Clemente, Anna Pau, Ilaria Galliano, Massimiliano Bergallo

**Affiliations:** 1 Early Infancy Special Care Unit, Regina Margherita Children Hospital, A.O.U. Città della Salute e della Scienza di Torino, 10126 Torino, Italy; francesco.savino@unito.it (F.S.); allegra.sardo@gmail.com (A.S.); 2 Department of Public Health and Pediatric Sciences, Immunopathology Laboratory, Medical School, University of Turin, 10126 Turin, Italy; gambarino.stefano@gmail.com (S.G.); maddalena.dini@unito.it (M.D.); anna.clemente424@edu.unito.it (A.C.); anna.pau@edu.unito.it (A.P.); ilaria.galliano@unito.it (I.G.)

**Keywords:** leptin, leptin receptor, polymorphisms, preterm birth, infants, genetics, parents, buccal swab

## Abstract

It has been proven that single-nucleotide polymorphisms (SNPs) in LEP and LEPR genes could predispose individuals to an increased risk of pregnancy adverse outcomes (PAOs) such as recurrent pregnancy loss (RPL) and pre-eclampsia. Preterm birth (PTB) is the leading cause of infant mortality. We decided to investigate the correlation between PTB and LEP and LEPR SNPs. The study cohort included families who underwent spontaneous PTB and control samples of families who had at-term-born (≥37 weeks of gestational age) children. Swabs were performed by rubbing the sticky end for about 30 s on the gum and on the inside of the cheek, allowing us to collect the flaking cells of the oral mucosa. Genotyping of the three SNPs—LEPRA668G, LEPG2548A and A19G—was carried out via an ARMS-MAMA real-time PCR procedure, as previously described. Regarding LEPG2548A, we found that the most expressed genotype in infants both in the preterm and the at-term group was AG; however, we did not discover any statistically significant difference (*p* = 0.97). Considering LEPA19G, none among the infants and parents were found to carry the AA genotype. No statistically significant differences were found between children, mothers and fathers belonging to preterm and at-term groups. We did not find a statistically significant association in newborns and their mother, but our results show a statistical correlation with the LEPRA668G genotype GG of the father. This fact can contribute to defining genetic risk factors for PTB. Further studies are certainly needed to better clarify the role of genetics in influencing preterm delivery.

## 1. Introduction

To date, leptin (LEP), a polypeptide hormone made of 167 amino acids, is mainly known for its role in regulating hunger and satiety mechanisms [1,2]. However, numerous scientific studies have demonstrated that this adipokine is implicated in the control of many pathways, including bone and cartilage growth, the immune system and the systemic inflammatory response, acting thus as a “pleiotropic” molecule [3]. What is more, LEP, which is primarily released by adipose tissue but also by the placenta [4], seems to play an important role in reproductive functions (such as oocyte maturation, embryo development and placentation) and pregnancy [5]. In fact, it has been shown that serum LEP levels increase significantly during gestation and decrease after birth, demonstrating an important role of LEP in childbearing [6]. In addition, as described by Childs et al. leptin-deficient mice are infertile, and the administration of exogenous LEP is able to restore fertility [7]. LEP exerts its actions through its receptor, LEPR, a single transmembrane protein made of 874 amino acids, expressed in the brain and peripheral tissues as kidneys, lungs, stomach, endometrium, placenta and umbilical cord [8,9]. Not only LEP alterations but also LEPR ones seem to have repercussions for reproduction and pregnancy; Pérez-Pérez et al. theorized that LEP and LEPR anomalies could be implicated in the pathogenesis of recurrent miscarriage, pre-eclampsia and intrauterine growth restriction [4]. It has been proven that single-nucleotide polymorphisms (SNPs) in LEP and LEPR genes could predispose individuals to these conditions; in particular, LEP G2548A (rs7799039) and LEPR A668G (rs1137101) are the most studied SNPs, and they have been shown to be associated with an increased risk of pregnancy adverse outcomes (PAOs) such as recurrent pregnancy loss (RPL) and pre-eclampsia [10]. Preterm birth (PTB), defined as the birth of a child before 37 completed weeks’ gestation, is the leading cause of death and disability in children under 5 years of age worldwide [11], but the exact pathogenesis is unknown. Whilst PTB is becoming a preventable condition for a very small subset of women, global rates of PTB continue to rise [12]. Recent estimates report that PTB affects approximately 11% of all livebirths, or approximately 15 million PTB per year [12]. Twin studies have also suggested that genetics account for 17–36% of PTB risk [13]. In attempting to understand the heritability of PTB, both maternal and fetal genetics have been assessed. It has been suggested that maternal genetics are responsible for 22.8% of the variations in gestational age in spontaneous births, with fetal genetics responsible for 12.7% of the variation [13]. Concerning metabolic and biosynthetic pathway-related polymorphisms, sustaining metabolic homeostasis is vital for embryonic development and survival. Polymorphisms within genes responsible for the biosynthesis and metabolism of fatty acids, lipoproteins, triglycerides and cholesterol have been associated with PTB in women and infants of various ethnicities [14]. Salem et al., in 2016, investigated the possible correlation between LEP and LEPR SNPs and PTB; they found that women and neonates bearing the homozygous mutated AA form of the LEP *G2548A* genotype had an increased risk of PTB, and women carrying LEPR A668G AA or AG genotypes had a four-fold increased risk for severe (gestational age ≤ 32 weeks) PTB [14]. Due to the suggested role of LEP and LEPR in pregnancy and ambiguous results, we decided to investigate the correlation between PTB and LEP and LEPR SNPs. On the basis of the implication of SNPs (LEP A19G, rs2167270) in the leptin levels in the first 6 month of life [2], we evaluated also the possible role of these SNPs on PTB, and we decided to extend genetic analysis also to the fathers of the babies; what is more, we performed genetic analysis not on blood but on buccal swab samples, and we determined LEP and LEPR polymorphisms through the procedure created by Bergallo et al., an amplification refractory mutation system-mismatch amplification mutation assay (ARMS-MAMA) real-time PCR [15].

## 2. Results

### 2.1. Characteristics of the Study Population

During the study period, 165 maternal, paternal and fetal swab samples were collected. Since one maternal and five paternal samples were lost, genetic analyses were performed on 159 subjects in total. The preterm group accounted for 54 subjects (18 infants + 36 parents), while the at-term one comprised 35 babies and 70 parents (105 in total). The former group included babies with a gestational age comprised between 26 and 36 + 6. For each child, an anamnestic form was filled out, registering infant as well as parents’ personal data, course of pregnancy, type of delivery and major illness. The main characteristics of the involved subjects, in relation to which the two groups resulted homogeneous (*p* > 0.05), are highlighted in Table 1.

### 2.2. Allelic Frequencies

Regarding LEPG2548A, A allele was the most frequent among preterm and at-term infants, without showing, however, a statistically significant difference (*p* = 0.84); on the other hand, G allele resulted as the most common among both mothers and fathers of the two groups, with no statistically significant difference (*p* = 0.83). Considering LEPA19G, G allele appeared to be the most represented among babies and their parents in the preterm and at-term groups; again, the difference was not statistically relevant, with a *p* value of 1 for children, 0.16 for mothers and 0.64 for fathers. At last, we analyzed LEPRA668G allelic frequencies, and we found that A allele was the most expressed; however, a non-significant difference was found among preterm and at-term children, as well as among parents (Table 2).

### 2.3. Genotypic Frequencies

Regarding LEPG2548A, we found that the most expressed genotype in infants both in the preterm and the at-term group was AG; however, we did not discover any statistically significant difference (*p* = 97). The mothers of the preterm babies turned out to be mainly carriers of AG genotype, while the mothers of at-term children had the same frequency of GG and AG genotypes (39%), but no significant difference was discovered among the two groups (*p* = 0.9), as well as for the fathers (*p* = 0.95), whose most detected genotype was AG. Considering LEPA19G, none among the infants and parents were found to carry the AA genotype. No statistically significant differences were found between children, mothers and fathers belonging to the preterm and at-term groups: preterm and at-term infants’ most common genotype was GG, preterm mothers’ was AG, while the at-term mothers’ was GG; on the other hand, preterm fathers turned out to be mainly carriers of GG genotype, and at-term fathers of the AG genotype. Finally, we analyzed LEPRA668G genotypic frequencies, and again, we did not find any statistically significant result. In preterm babies, the most common genotype was AG; in at-term babies, AA. Regarding parents, the mothers of the two groups both showed the AG genotype as the most frequent, while preterm and at-term fathers showed the AA genotype (Table 3).

### 2.4. Association between Neonates and Parents Polymorphism and Preterm Births

We evaluated if there were any differences between mutated homozygotes and wild-type homozygotes + heterozygotes among preterm and at-term groups for LEPG2548A and LEPRA668G. We did not find any statistically significant differences between the LEPG2548A genotypes analyzed, as shown in Table 4. On the contrary, we found statistically significant differences in LEPRA668G genotypes. GG LEPRA668G genotypes were more frequent in fathers in the preterm group. Since none of the subjects turned out to be carriers of the mutated homozygous (AA) genotype for LEPA19G, we did not consider LEPA19G in this analysis.

## 3. Discussion

Over the past two decades, there have been significant technological advances that have improved our ability to obtain genetic data and address challenges in disease prevention and treatment. Studies have been conducted to improve our understanding of the biological mechanisms underlying PTB and to translate research findings into a clinical setting. The development of prognostic tools that stratify treatment to enable targeted and personalized PTB prevention has been a major focus of many genetic studies on PTB. PTBs occur at less than 37 weeks’ gestational age, accounting for 75% of perinatal mortality. Up to now, the preterm birth rate has risen dramatically in most industrialized countries; it is estimated to be around 12–13% in the USA and 5–9% in Europe [13,16]. Despite its high incidence, the exact pathogenesis of preterm delivery is still not known. A genetic component seems to play a crucial role; in particular, variations in the maternal genome are thought to be the most implicated in influencing the course of pregnancy; however, recently, it has been proposed that fetal genetic variations might also contribute to childbirth outcome [17,18]. For this reason, based on the results obtained by Salem et al., we wanted to further investigate the possible impact of LEP and LEPR polymorphisms on preterm delivery [14]. It is, in fact, confirmed that LEP and its receptor are involved in the regulation of reproductive functions, as demonstrated by the fact that LEP levels rise significantly during the first gestational trimester along with estrogen and β-hCG, as well as the fact that LEP and LEPR transcripts are expressed early in trophoblast during pregnancy, proving the paracrine and autocrine actions of this adipokine [5,19,20,21]. The association between serum LEP levels and PAOs has been investigated by many authors, and it has been proven that variations in LEP levels significantly increase the risk of PAO; in particular, the higher the serum LEP values are, the greater the risk of PTB seems to be, as theorized by Vàzquez et al. and Strobel et al. [22,23]. LEP and LEPR polymorphisms are known to be associated with altered serum LEP levels; for example, Marcello et al. found that the carriers of the homozygote mutant AA genotype for LEPG2548A have higher plasma LEP levels in comparison to carriers of AG and GG genotypes [24,25]. It is not totally clear through which pathways LEP could lead to PTB; however, it seems that augmented LEP levels are associated with an increase in sympathetic activity, in mitochondrial superoxide synthesis and with an activation of matrix metalloproteinases which modulate the vascular structures; all these conditions are believed to generate oxidative stress as well as endothelial and placental dysfunction [26]. Leptin is a hormone that regulates peripheral energy stores and is therefore mainly found in adipose tissue. It is known that there is a positive correlation between adipose tissue and LEP levels. In pregnant women, LEP levels increase in the first trimester, even before there is a noticeable increase in body weight, suggesting that factors other than increased adiposity influence LEP levels. During pregnancy, the placenta is an additional source of LEP, which also plays a crucial role in cell proliferation, implantation and fetal growth. LEP concentration in cord blood has been found to correlate strongly with placental and birth weight [14]. LEPs from the placenta pass into the maternal blood and contribute to maternal LEP blood levels. In fact, most LEPs are expressed in the placenta, entering the maternal serum and increasing the maternal LEP serum level. Thus, the embryo has a major influence on the LEP serum level of its mother. In this context, our new findings regarding neonatal genetic variation as a factor in PTB are important. Salem et al. show that the effect of LEP polymorphism on PTB is strong and significant and it is sufficient that either the mother or her respective neonate independently carry the LEP homozygote mutant AA genotype to markedly increase the risk for PTB [14].

As opposed to Salem et al., we did not find any statistically significant correlation between LEP and LEPR polymorphisms in infants and mothers and PTB. Since LEPG2548A is thought to be in linkage disequilibrium with the less-studied LEPA19G, we also evaluated this latter SNP [15,27,28]; interestingly, none of the subjects turned out to be carriers of the AA genotype; what is more, the frequency of the A allele was lower than that of the G allele in children, mothers and fathers all; given the scarce literature on this SNP, which is mainly studied in relation to neoplastic pathologies, the implication of these data remains to be clarified [29].

We decided, also, to involve the fathers in our study of the babies since, although childbirth is strictly dependent on the mother–fetus binomial, the genetic material of the embryo derives half from the paternal contribution [30,31]. In this contest, considering that Salem et al. found a positive correlation between PTB and the homozygous mutated AA genotype for LEPG2548A in children, we wanted to evaluate the risk of the infant inheriting the mutated allele from the father and therefore being exposed to an increased probability of PTB [32].

We find a statistically significant difference among the preterm and at-term groups concerning the homozygote mutant LEPRA668G genotype GG reflected in the 3.6-fold increased risk of preterm birth.

Also, the LEPRA668G genotype seems to be involved in plasma levels of leptin [2], and this fact could be associated with oxidative stress as well as endothelial and placental dysfunction [26]. Single-nucleotide polymorphisms (SNPs) of LEPR A668G (Q223R, rs1137101) have been certified to affect the binding activity of LEPR and leptin, ultimately changing the leptin signaling. The SNP have been reported to be associated with a variety of diseases, such as type 2 diabetes mellitus (T2DM), polycystic ovary syndrome (PCOS), coronary artery disease, essential hypertension, nonalcoholic fatty liver disease and endometrial cancer [33]. PTB etiology is multifactor, with a strong genetic component. We do not attempt any correspondence with previous data reported by Salem but add an actor (father) in the importance of these LEP and LEPR SNPs.

Regarding the procedure of DNA extraction, we choose to perform it on the less invasive buccal swab, which is also less painful than blood sampling. It is, in fact, confirmed that repetitive hurting stimulations in infants, also in very young ones, can induce changes in the nervous system, both at the peripheral and central levels, with alterations of the pain threshold [34,35]. The swab allows for the collection of exfoliation cells of the oropharynx, and, since the DNA is the same in any cell of an individual, the obtained genetic material can be used in the same way as a blood sample [36]; it has been seen, in fact, that the results of the DNA extraction from different types of biological material are comparable and have the same degree of accuracy [37,38]. In our experience, this method significantly increased the compliance of the parents in subjecting the child to the procedure of sampling. The main limitations of this study are related to the available sample size, which does not allow for any generalization of our results. We use of the “late preterm” gestational age of many members of the preterm group (around 36 weeks), so it might have been more useful for the purposes of this study to consider a population with a lower gestational age to better highlight any differences between the preterm and at-term groups.

Up to date, it has been proven that LEP is implicated in the pathophysiology of many events, even if its function is often underestimated [39]. To the best of our knowledge, this is the second study evaluating a possible correlation between LEP and LEPR polymorphisms and PTB, but the first to also involve the fathers of the babies with the aim to investigate the possible role of paternal genetic contribution on delivery outcomes. PTB is a global problem in obstetrics, affecting nearly 15 million babies worldwide every year. The development of PTB is multifactorial and is influenced by both environmental and genetic factors. There is a great deal of research looking at the genetics of PTB, with a focus on identifying the genetic polymorphisms associated with it. Although investigating the genetics of PTB is challenging because the PTB population is highly heterogenous, we did not find a statistically significant association between newborns and their mother, but our results show a statistical correlation with the LEPRA668G genotype GG of the father. This fact can contribute to defining the genetic risk factors for PTB. Further studies are certainly needed to better clarify the role of genetics in influencing preterm delivery.

## 4. Materials and Methods

### 4.1. Study Desig and Subjects

We included in our prospective case-control study 96 infants admitted at “Terapia Subintensiva Allargata Prima Infanzia”, University Division, Regina Margherita Children Hospital, Città della Salute e della Scienza di Torino, between October 2018 and March 2019. Exclusion criteria were mothers who experienced complicated births or suffered from pre-eclampsia. The study cohort included families who underwent spontaneous PTB and control samples of families who had at-term-born (≥37 weeks of gestational age) children. Among the 96 initially included infants, only the parents of 55 children permitted their enrollment in the study via written informed consent. For each baby, we recruited both father and mother, with a total of 165 subjects who underwent a buccal swab for DNA extraction.

### 4.2. Buccal Swab Sampling

Swabs were performed by rubbing the sticky end for about 30 s on the gum and on the inside of the cheek, allowing us to collect the flaking cells of the oral mucosa; they were then placed in a special test tube, without liquid or culture medium, and stored in the refrigerator (at −80 °C) before the extraction procedure.

### 4.3. DNA Extraction and Genotyping Description

Genomic DNA was extracted from the swabs using the Maxwell16 LEV Blood DNA kit with automatic extractor Maxwell16 System (Promega, Madison, WI, USA), following the manufacturer’s instructions. Genotyping of the three SNPs—LEPR A668G, LEP G2548A and A19G—was carried out via an ARMS-MAMA real-time PCR procedure, as previously described by the authors [15]. These assays have been the subject of technology transfer in favor of the academic spin-off of the University of Turin BioMole (BioMole srl. Turin, Italy) G2548A-LEP rs7799039 PP-BioMole-033, G19A-LEP rs2167270 PP-BioMole-034 and Q223R-LEPR rs1137101 PP-BioMole-035, and they are now commercially available. In the ARMS-PCR assay, the specificity of forward or reverse primers is given by the terminal 3′ nucleotide, while the MAMA-PCR improves the discrimination power of the allele-specific primers by adding a mismatched nucleotide near the 3′ terminal region. Therefore, the ARMS-MAMA method allows us to greatly reduce the background noise and it is, thus, a reliable and easily interpretable technique, without needing to process the post-amplification sample as is occurred but instead in the PCR-RFLP (restriction fragment length polymorphism) procedure.

### 4.4. Statistical Analysis

Data analyses were conducted on the total sample using the statistical application GraphPad Prism (version 5). Sample size was evaluated by power analysis. Demographic data, as well as information on pregnancy, delivery and the neonatal period were presented as descriptive statistics. Continuous variables were expressed as means and standard deviations. For each SNP, the allele frequencies were computed via simple counting, and the Hardy–Weinberg equilibrium was tested with Fisher’s exact test. Bivariate analyses to compare categorical variables were performed using Fisher’s and Chi-square statistical tests, with independent samples *t*-test to compare quantitative variables. All tests were conducted in two sails, with a fixed significance α = 5% (*p* = 0.05).

## Figures and Tables

**Table 1:** Demographic description of the studied cohort; values expressed as mean (+/− standard deviation).

Characterization	Preterm Group (n = 54)	At Term Group (n = 105)	*p*-Value
Gender of newborn % (male)	38%	50%	0.39
Mean maternal age at birth, years	32 (5.03)	33.6 (4.19)	0.26
Mean paternal age at birth, years	35.6 (4.72)	39.9 (4.43)	0.94
Mean gestational age at delivery, weeks	34.1 (2.87)	39.3 (2.09)	0.57
Mean number of pregnancies	2.28 (1.07)	1.22 (0.98)	0.86
Mean number of live births	2.43 (1.75)	1.72 (1.03)	0.74
Abortions	0.10 (0.68)	0.16 (0.93)	0.93
Caesarean sections	0.40 (1.02)	0.25 (0.65)	0.09

**Table 2:** LEPG2548A, LEPA19G and LEPRA668G allelic frequencies in preterm and at-term groups.

	SNP Subjects	Allelic Frequencies in Preterm Group (%)		Allelic Frequencies in At-Term Group (%)		*p*-Value
		G	A	G	A	
LEP G2548A	Infants	45	55	42	58	0.84
	Mothers	56	44	58	42	0.83
	Fathers	59	41	56	44	0.83
LEP A19G	Infants	77	23	76	24	1.00
	Mothers	67	33	79	21	0.16
	Fathers	76	24	71	29	0.64
LEPR A668G	Infants	40	60	33	67	0.54
	Mothers	42	58	39	61	0.83
	Fathers	35	65	27	73	0.75

**Table 3:** LEPG2548A, LEPA19G and LEPRA668G genotypic frequencies in preterm and at-term groups.

	SNP Subjects	Genotypic Frequencies in Preterm Group (%)			Genotypic Frequencies in At-Term Group (%)			*p*-Value
		GG	AG	AG	GG	AA	AG	
LEP G2548A	Infants	15	25	60	11	28	61	0.97
	Mothers	33	22	45	39	22	39	0.90
	Fathers	35	18	47	33	21	46	0.95
LEP A19G	Infants	55	-	45	53	-	47	1.00
	Mothers	33	-	67	58	-	42	0.68
	Fathers	53	-	47	42	-	58	0.55
LEPR A668G	Infants	15	35	50	12	44	44	0.77
	Mothers	6	27	67	11	28	61	0.75
	Fathers	18	47	35	6	52	42	0.42

**Table 4:** Mutated homozygous versus (wild-type homozygous + heterozygous) genotypic frequencies for LEPG2548A and LEPRA668G in preterm and at-term groups.

	SNP Subjects	Preterm Group (%)		Term Group (%)		*p*-Value
		**AA**	**AG+GG**	**AA**	**AG+GG**	
LEP G2548A	Infants	26	74	28	72	1.00
	Mothers	22	78	22	78	1.00
	Fathers	18	82	21	79	0.92
		**GG**	**AG+AA**	**GG**	**AG+AA**	
LEPR A668G	Infants	16	84	11	89	0.75
	Mothers	11	89	6	94	0.31
	Fathers	18	82	6	94	0.0153 * * statistically significant

The father carrying the homozygote mutant GG LEPRA668G genotype markedly (odd ratio: 3.6-fold) increases the risk of PTB.

## Data Availability

The data presented in this study are available on request from the corresponding author. The data are not publicly available due to privacy.

## References

[1] Seoane-Collazo P., Martínez-Sánchez N., Milbank E., Contreras C. (2020). Incendiary Leptin. Nutrients.

[2] Savino F., Sardo A., Rossi L., Benetti S., Savino A., Silvestro L. (2016). Mother and Infant Body Mass Index, Breast Milk Leptin and Their Serum Leptin Values. Nutrients.

[3] Pérez-Pérez A., Vilariño-García T., Fernández-Riejos P., Martín-González J., Segura-Egea J.J., Sánchez-Margalet V. (2017). Role of leptin as a link between metabolism and the immune system. Cytokine Growth Factor Rev..

[4] Pérez-Pérez A., Toro A., Vilariño-García T., Maymó J., Guadix P., Dueñas J.L., Fernández-Sánchez M., Varone C., Sánchez-Margalet V. (2018). Leptin action in normal and pathological pregnancies. J. Cell. Mol. Med..

[5] Catteau A., Caillon H., Barrière P., Denis M.G., Masson D., Fréour T. (2016). Leptin and its potential interest in assisted reproduction cycles. Hum. Reprod. Update.

[6] Xiao W.Q., He J.R., Shen S.Y., Lu J.H., Kuang Y.S., Wei X.L., Qiu X. (2020). Maternal circulating leptin profile during pregnancy and gestational diabetes mellitus. Diabetes Res. Clin. Pract..

[7] Childs G.V., Odle A.K., MacNicol M.C., MacNicol A.M. (2021). The Importance of Leptin to Reproduction. Endocrinology.

[8] Dam J. (2018). Trafic et signalisation du récepteur de la leptine [Traffic and signalisation of the leptin receptor]. Biol. Aujourdhui.

[9] Srinivasan G., Parida S., Pavithra S., Panigrahi M., Sahoo M., Singh T.U., Madhu C.L., Manickam K., Shyamkumar T.S., Kumar D. (2021). Leptin receptor stimulation in late pregnant mouse uterine tissue inhibits spontaneous contractions by increasing NO and cGMP. Cytokine.

[10] Saad A., Adam I., Elzaki S.E.G., Awooda H.A., Hamdan H.Z. (2020). Leptin receptor gene polymorphisms c.668A>G and c.1968G>C in Sudanese women with preeclampsia: A case-control study. BMC Med. Genet..

[11] Perin J., Mulick A., Yeung D., Villavicencio F., Lopez G., Strong K.L., Prieto-Merino D., Cousens S., E Black R., Liu L. (2022). Global, regional, and national causes of under-5 mortality in 2000–19: An updated systematic analysis with implications for the Sustainable Development Goals. Lancet Child Adolesc. Health.

[12] Chawanpaiboon S., Vogel J.P., Moller A.-B., Lumbiganon P., Petzold M., Hogan D., Landoulsi S., Jampathong N., Kongwattanakul K., Laopaiboon M. (2019). Global, regional, and national estimates of levels of preterm birth in 2014: A systematic review and modelling analysis. Lancet Glob. Health.

[13] Mead E.C., Wang C.A., Phung J., Fu J.Y., Williams S.M., Merialdi M., Jacobsson B., Lye S., Menon R., Pennell C.E. (2023). The Role of Genetics in Preterm Birth. Reprod. Sci..

[14] Salem H., Rosenfeld T., Altarescu G., Grisaru-Granovsky S., Birk R. (2016). Maternal and neonatal leptin and leptin receptor polymorphisms associated with preterm birth. Gene.

[15] Savino F., Rossi L., Di Stasio L., Galliano I., Montanari P., Bergallo M. (2016). Mismatch Amplification Mutation Assay Real-Time PCR Analysis of the Leptin Gene G2548A and A19G Polymorphisms and Serum Leptin in Infancy: A Preliminary Investigation. Horm. Res. Paediatr..

[16] Purisch S.E., Gyamfi-Bannerman C. (2017). Epidemiology of preterm birth. Semin. Perinatol..

[17] York T.P., Eaves L.J., Neale M.C., Strauss J.F. (2014). The contribution of genetic and environmental factors to the duration of pregnancy. Am. J. Obstet. Gynecol..

[18] Yılmaz Y., Verdi H., Taneri A., Yazıcı A.C., Ecevit A.N., Karakaş N.M., Tarcan A., Haberal A., Ozbek N., Atac F.B. (2012). Maternal-fetal proinflammatory cytokine gene polymorphism and preterm birth. DNA Cell Biol..

[19] Henson M.C., Castracane V.D. (2006). Leptin in pregnancy: An update. Biol. Reprod..

[20] Sattar N., Greer I.A., Pirwani I., Gibson J., Wallace A.M. (1998). Leptin levels in pregnancy: Marker for fat accumulation and mobilization?. Acta Obstet. Gynecol. Scand..

[21] Schanton M., Maymó J., Camisay M.F., Pérez-Pérez A., Casale R., Sánchez-Margalet V., Erlejman A., Varone C. (2020). Crosstalk between estradiol and NFκB signaling pathways on placental leptin expression. Reproduction.

[22] Vázquez M.J., Romero-Ruiz A., Tena-Sempere M. (2015). Roles of leptin in reproduction, pregnancy and polycystic ovary syndrome: Consensus knowledge and recent developments. Metabolism.

[23] Strobel A., Issad T., Camoin L., Ozata M., Strosberg A.D. (1998). A leptin missense mutation associated with hypogonadism and morbid obesity. Nat. Genet..

[24] Marcello M.A., Calixto A.R., de Almeida J.F., Martins M.B., Cunha L.L., Cavalari C.A., Etchebehere E.C., da Assumpção L.V., Geloneze B., Carvalho A.L. (2015). Polymorphism in LEP and LEPR May Modify Leptin Levels and Represent Risk Factors for Thyroid Cancer. Int. J. Endocrinol..

[25] Savino F., Sardo A., Montanari P., Galliano I., Di Stasio L., Bergallo M., Silvestro L. (2017). Polymorphisms in Lep and Lepr Genes in Infants: Correlation with Serum Leptin Values in the First 6 Months of Life. J. Am. Coll. Nutr..

[26] Poston L. (2002). Leptin and preeclampsia. Semin. Reprod. Med..

[27] Hui T.J., Burt A. (2020). Estimating linkage disequilibrium from genotypes under Hardy-Weinberg equilibrium. BMC Genet..

[28] Lucantoni R., Ponti E., Berselli M.E., Savia G., Minocci A., Calò G., de Medici C., Liuzzi A., Di Blasio A.M. (2000). The A19G polymorphism in the 5’ untranslated region of the human obese gene does not affect leptin levels in severely obese patients. J. Clin. Endocrinol. Metab..

[29] Llanos A.A., Brasky T.M., Mathew J., Makambi K.H., Marian C., Dumitrescu R.G., Freudenheim J.L., Shields P.G. (2014). Genetic variation in adipokine genes and associations with adiponectin and leptin concentrations in plasma and breast tissue. Cancer Epidemiol. Biomark. Prev..

[30] Du Fossé N.A., van der Hoorn M.P., van Lith J.M.M., le Cessie S., Lashley E.E.L.O. (2020). Advanced paternal age is associated with an increased risk of spontaneous miscarriage: A systematic review and meta-analysis. Hum. Reprod. Update.

[31] Bengtson M.B., Solberg I.C., Aamodt G., Jahnsen J., Moum B., Vatn M.H., IBSEN Study Group (2010). Relationships between inflammatory bowel disease and perinatal factors: Both maternal and paternal disease are related to preterm birth of offspring. Inflamm. Bowel. Dis..

[32] Tullius Z., Rankin K., DeSisto C., Collins J.W. (2020). Adverse birth outcome across the generations: The contribution of paternal factors. Arch. Gynecol. Obstet..

[33] Zeng S., Wu Y., Zhou M., Bai H., Liu X., Fan P. (2023). Association between genetic polymorphisms of leptin receptor and preeclampsia in Chinese women. J. Matern. Fetal Neonatal Med..

[34] Walker S.M. (2013). Biological and neurodevelopmental implications of neonatal pain. Clin. Perinatol..

[35] Hohmeister J., Kroll A., Wollgarten-Hadamek I., Zohsel K., Demirakça S., Flor H., Hermann C. (2010). Cerebral processing of pain in school-aged children with neonatal nociceptive input: An exploratory fMRI study. Pain.

[36] Xu Y.L., Zhou H.Y., Xu J., Liu N.P. (2012). Use of buccal swab as a source of genomic DNA for genetic screening in patients with age-related macular degeneration. Chin. J. Ophthalmol..

[37] Tang Z., Tang X., Xue L., Guan M. (2021). A non-invasive method for detecting mitochondrial tRNAThr15927G>A mutation. J. South. Med. Univ..

[38] Mesman A.W., Calderon R.I., Pollock N.R., Soto M., Mendoza M., Coit J., Zhang Z., Aliaga J., Lecca L., Holmberg R.C. (2020). Molecular detection of Mycobacterium tuberculosis from buccal swabs among adult in Peru. Sci. Rep..

[39] Zhang Y., Chua S. (2017). Leptin Function and Regulation. Compr. Physiol..

